# Editorial: Research on acculturation and education: current methodological approaches

**DOI:** 10.3389/fpsyg.2023.1321439

**Published:** 2023-11-07

**Authors:** Elena Makarova, Nanine Lilla, Christine Wolfgramm, Dina Birman

**Affiliations:** ^1^Institute for Educational Sciences, University of Basel, Muttenz, Switzerland; ^2^Department of Education and Psychology, Freie Universität Berlin, Berlin, Germany; ^3^Education Secondary Level I & Centre for Teaching Professions and Continuing Professional Development, Zurich University of Teacher Education, Zurich, Switzerland; ^4^School of Education and Human Development, University of Miami, Coral Gables, FL, United States

**Keywords:** acculturation, school context, education, measurement, methods

Acculturation refers to the process of change that occurs when people of different cultural backgrounds are in enduring first-hand contact (Redfield et al., 1936). This process can manifest itself in a stronger or weaker orientation toward both cultures. Berry's (1997) conceptualization of four different patterns of acculturation orientation—integration, assimilation, separation/segregation, and marginalization—is well-established in the field of cross-cultural psychology and beyond.

Decades of acculturation research have led to an extensive body of literature on acculturation and its outcomes in terms of psychological and sociocultural adjustment. For children and youth, research has especially shown relationships of acculturation and different educational outcomes (e.g., Trickett and Birman, 2005; Makarova and Birman, 2015, 2016; Lilla et al., 2021; Makarova et al., 2021; Thürer et al., 2023). For teachers, research has shown that teachers' acculturation attitudes are related to their professional competencies and that teachers play key role in migrant students' school adjustment (e.g., Makarova and Herzog, 2013; Makarova et al., 2019). Furthermore, there is evidence suggesting, that (mis)matching constellations of acculturation orientations of immigrant students' and teachers' impact students' school adjustment (Birman and Tran, 2017; Haenni Hoti et al., 2019).

Acculturation research has also brought forward a variety of conceptual and methodological approaches for the empirical investigation of acculturation. In addition, a multitude of measures and analytical methods increases the various approaches to capturing the acculturation construct (Sam and Ward, 2021).

This Research Topic brings together a collection of six original papers that delve into the intricate relationship between *acculturation processes and educational outcomes*. The papers employ a *range of methodologies*, from quantitative analyses to mixed-methods approaches, offering a comprehensive view of the Research Topic. This methodological diversity enriches the broader understanding of acculturation and education. Moreover, this Research Topic spans research across multiple cultural contexts including Germany, Italy, Turkey, Switzerland, and South Korea. The aim of this editorial is to frame the objectives and findings of these papers within a broader academic discourse, thereby providing a cohesive understanding of the current state of research in this field.

The study by Ulbricht et al. focuses on the *acculturation attitudes of teachers*, primarily representing mainstream culture in culturally diverse schools. It argues that norms of cultural pluralism and equality are directly and positively associated with facets of teachers' intercultural self-efficacy. The study by Auer et al. also focuses on teachers' acculturation orientations and investigates its role in the transmission of values to primary school pupils. Although teachers' acculturation orientations did not play a role in predicting pupils' values, teachers were found to be crucial value transmission agents.

The study by Sidler focuses on mutual *acculturation attitudes among adolescents* in the school context. The study reveals three distinct acculturation profiles: mutual integration, multiculturalism, and cultural distancing. It finds that students in the cultural distancing profile are more likely to identify as not having a migration background compared to those in the mutual integration profile. In the same vein, the study by Lilla employs a person-centered approach and delves into the acculturation patterns of immigrant students and finds that the integration and marginalization profiles are the least common among immigrants, but the distribution varies for instance depending on the group of origin.

The study by Jin et al. focuses on *international students* and finds that bicultural self-efficacy fully mediates the relationship between acculturation to mainstream culture and career decision-making self-efficacy. Similarly, the study by Nazir and Özçiçek investigates the adjustment challenges of female international university students. It shows that these students experience challenges in academic, sociocultural, and personal dimensions of adjustment with the sociocultural domain as the most challenging one. It reveals that the students encountered various problems ranging from cultural differences to discrimination and often indicated maladaptive or dysfunctional coping strategies.

Overall, this Research Topic offers a multifaceted exploration of acculturation within educational contexts. The findings underscore that the acculturation process in educational settings involves minority and majority students and teachers alike highlighting that school itself functions as an acculturative agent.

Finally, this Research Topic points to several new directions for further research in the field of acculturation and education:

- Several papers employ different theoretical frameworks for understanding acculturation. Future research could aim to *integrate these theoretical models* to provide a more comprehensive understanding of acculturation in educational settings.
- While some papers focus on specific demographic groups, there is room for more research that considers *intersectionality*—how various social identities (e.g., race, gender, socioeconomic status) intersect to impact acculturation experiences in educational settings.
- Most of the papers employ cross-sectional designs. *Longitudinal studies* could provide insights into how acculturation attitudes and experiences evolve over time, especially in relation to educational outcomes.
- The papers focus on various geographical contexts. *Comparative studies* could provide insights into how cultural and national contexts influence acculturation processes in education.
- While the papers discuss individual and group experiences, there is a gap in the examination of *how educational policies impact acculturation processes*. Future research could explore the role of policies in facilitating or hindering acculturation in schools.
- In an increasingly digital world, the role of *technology in acculturation* within educational settings remains underexplored. Research could focus on how digital platforms and online education impact acculturation experiences.

In summary, while the current Research Topic provides valuable insights into the relationship between acculturation and education, it also opens up multiple avenues for further exploration. These new directions could significantly enrich the academic discourse and have practical implications for educators, policymakers, and researchers alike.

## Author contributions

EM: Conceptualization, Writing—original draft. NL: Writing—review & editing. CW: Writing—review & editing. DB: Writing—review & editing.
